# Efficacy of small back optic zone design on myopia control for corneal refractive therapy (CRT): a one-year prospective cohort study

**DOI:** 10.1186/s40662-023-00364-z

**Published:** 2023-11-20

**Authors:** Xuewei Li, Lili Zuo, Heng Zhao, Jie Hu, Tao Tang, Kai Wang, Yan Li, Mingwei Zhao

**Affiliations:** 1 Institute of Medical Technology, Peking University Health Science Center, Beijing, China; 2 Department of Ophthalmology and Clinical Centre of Optometry, Peking University People’s Hospital, Beijing, 100044 China; 3 College of Optometry, Peking University Health Science Center, Beijing, China; 4 Eye Disease and Optometry Institute, Peking University People’s Hospital, Beijing, China; 5 Beijing Key Laboratory of the Diagnosis and Therapy of Retinal and Choroid Diseases, Beijing, China

**Keywords:** Back optic zone diameter, Myopia control, Orthokeratology, Treatment zone, Corneal higher-order aberrations

## Abstract

**Background:**

To investigate the control effect on the axial length elongation using corneal refractive therapy (CRT) with different optical zone diameters (BOZDs). We also sought to compare the difference in higher-order aberrations (HOAs), treatment zone (TZ) size and Zernike defocus coefficient with different BOZDs and seek the optimal parameter for predicting axial elongation.

**Methods:**

This prospective cohort study included 7- to 14-year-olds fitted with orthokeratology (ortho-K) lenses of 5-mm (5-mm group) or 6-mm BOZD (6-mm group). Axial length (AL), corneal topography, HOAs and Zernike defocus coefficient were obtained at baseline, and 1, 3, 6, 9 and 12 months follow-up visits. Multivariate regression analyses were used to explore the association between AL change and ocular biometric parameters. Receiver operating characteristic (ROC) curve analysis was used to determine the best diagnostic value for AL change in ocular biometric parameters.

**Results:**

In total, 301 participants completed the one-year follow-up. The mean AL change in the 5-mm group (0.13 ± 0.18 mm) was less than that in the 6-mm group (0.27 ± 0.15 mm) at the 12 months visit. The TZ size and decentration were smaller, while the Zernike defocus coefficient and HOAs were higher in the 5-mm group (all *P* < 0.05). Older age and smaller TZ size were protective factors against AL elongation in multiple regression. In predicting AL elongation, TZ diameter yielded an area under the ROC curve of 0.684 with a cut-off value of 3.82 mm.

**Conclusion:**

The 5-mm group showed 0.14 mm (51.8%) less axial elongation than the 6-mm group. The 5-mm BOZD produced a smaller TZ size, higher Zernike defocus coefficient and higher HOA after reshaping of the cornea. TZ size was the best predictor of AL elongation. TZ diameter less than 3.82 mm may lead to AL elongation less than 0.2 mm in one year.

**Supplementary Information:**

The online version contains supplementary material available at 10.1186/s40662-023-00364-z.

## Background

Myopia is a refractive disorder that typically presents with abnormal axial length (AL) elongation during childhood, and various studies have shown that the global prevalence of myopia is increasing significantly [1–3]. Excessive axial elongation may lead to serious vision-threatening complications, which may burden public health and the economy [4]. Although its mechanism of myopia control remains unclear, orthokeratology (ortho-K) has been widely accepted as a major intervention in reducing myopia progression and axial elongation [5–8].

Changes in relative peripheral defocus [9] and higher-order aberrations (HOAs) have been hypothesized to explain the mechanism by which ortho-K slows myopia progression. In chick and monkey models, peripheral myopic defocus can reduce axial elongation [10, 11], especially in the near periphery approximately 20° from the fovea [12]. Many studies have attempted to assess the features of relative peripheral defocus, such as certain axis directions [13], specific regions [14], and the spatial distribution of relative corneal refractive power shift [15–17], and identify those that are related to AL elongation. Our recent study also used a mathematical method to quantify the morphology of the overall defocus characteristics [18], which can reflect the steeper or flatter mid-peripheral corneal power changes in three-dimensional space. An increased HOA may also play a role in the retardation of axial elongation in ortho-K [19]. After corneal reshaping, notable changes including total HOAs, spherical aberrations and coma [20–22] were observed, which are beneficial for slowing axial elongation.

Recently, a short-term study [23] reported that ortho-K designed with a smaller back optic zone diameter (BOZD) could produce greater peripheral defocus, higher HOAs and a smaller treatment size (TZ), while effective slowing of axial elongation was reported by some long-term follow-up studies [24, 25]. The TZ was defined as the area of the central cornea flattened by ortho-K [26]. To date, only a limited number of studies have examined whether the smaller BOZD of some lens designs contributes to AL retardation, and few have reported which variable (e.g., HOA, TZ size and peripheral defocus) can most effectively predict the progression of myopia.

Here, we investigated the effect of different BOZDs on myopia control, and compared the HOAs, TZ sizes and Zernike defocus coefficients of lenses with different BOZDs. Importantly, we attempted to identify the indicator that best predicts myopia progression and to find the optimal cut-off value of that variable to further improve myopia control for clinical ortho-K wearers.

## Methods

### Study design and participants

The study adhered to the Declaration of Helsinki and was approved by the Ethics Committee of the Peking University People’s Hospital (2021PHB386-001). Both the children and their parents signed a consent form after the study procedures and possible risks were explained.

This prospective study was conducted at the Peking University People’s Hospital Eye Center from October 2021 to February 2023. The inclusion criteria were as follows: (1) Chinese children aged 7 to 14 years old; (2) axial myopia between − 1.00 D and − 5.50 D; (3) myopic astigmatism < 1.50 D; and (4) best corrected monocular visual acuity equal to or better than 20/20; (5) initially diagnosed as myopia after visiting our center; (6) less than 1.00 D difference in spherical equivalent refraction (SER) between the two eyes. The exclusion criteria were as follows: (1) history of ocular or systemic conditions such as connective tissue disorders, Down syndrome, Marfan syndrome; (2) history of ocular surgeries or contact lens wearing; (3) prior myopia control treatment; (4) prescription modification during ortho-K lens wear; and (5) discontinuation of ortho-K lens wear during the study period.

### Lens fitting

Each participant was randomly assigned to be fitted with either a 6-mm BOZD (6-mm group) or a 5-mm BOZD (5-mm group) ortho-K lenses of the same type, corneal refractive therapy (CRT; Paragon Vision Sciences, USA), in both eyes. Lens fitting was performed by the same experienced clinician according to the manufacturer’s recommended guidelines. Subjects were required to wear the lenses for at least eight consecutive hours per night and at least 6 days per week. Study participants were scheduled to visit at baseline, 1 day, 1 week, 1 month, 3 months, 6 months, 9 months, and 12 months. Those who missed any of these visits were considered dropouts and excluded from the final analysis. At each visit, a complete eye examination was performed, including visual acuity (VA), slit-lamp examination, ocular biometrics and corneal topography. The subjects were measured between 8 a.m. and 12 p.m., and each follow-up visit was scheduled to approximate the time window of the first measurement to minimize diurnal variation [27, 28].

### Ocular parameter measurements

At baseline, cycloplegic refraction was measured with an autorefractor (KR-9000, Topcon, Japan). Cycloplegia was induced 30 min before refraction measurements with three drops of 0.5% tropicamide/0.5% phenylephrine instilled 5 min apart. The SER was calculated as the spherical power plus 1/2 the cylindrical power.

At the time of enrolment and 3 months, 6 months, 9 months, and 12 months after lens delivery, axial length (AL) was measured with an IOL Master 700 (Carl Zeiss Meditec, Oberkochen, Germany). At each visit, five consecutive measurements were collected, with intrasession differences no greater than 0.02 mm, and the average of the five measurements was used in further data analysis.

Corneal parameters were assessed at baseline and every follow-up visit by using the Sirius corneal topography system (CSO, Italy) to obtain the average K readings (Ave-K), e value and pupil diameter (PD). PD was measured under the same light intensity (photopic model, 40 lx) each time.

HOAs parameters were also obtained by the Sirius corneal topography system, which analyzed the corneal wavefront aberrations using Zernike polynomial analysis. All parameters are computed as the root mean square (RMS) and were taken over a 6.0 mm diameter. Parameters including total HOAs (from the 3rd to the 7th order terms), total coma (square root of the sum of the squared coefficients of Z(3 ± 1) and Z(5 ± 1)), total spherical aberration (SA) [square root of the sum of the squared coefficients of Z(4 ± 0) and Z(6 ± 0)] and Zernike coefficients from the 3rd to the 7th orders were analyzed.

### Evaluation of the Zernike defocus coefficient and TZ

The raw data (31 × 256, csv file) of the corneal tangential corneal power (at baseline and every follow-up visit) were exported from the Sirius system to evaluate the Zernike defocus coefficient and TZ. The differential topographical map was generated by minus tangential power map after wearing ortho-K from the baseline visit, which the following calculations are based on.

The Zernike defocus coefficient and TZ size were calculated using previously described methods [18, 29]. More specifically, the TZ size ($\pi \cdot {r}^{2}$) and Zernike defocus coefficient (${C}_{2}^{0}$, D) were calculated.

**Fig. 1:**
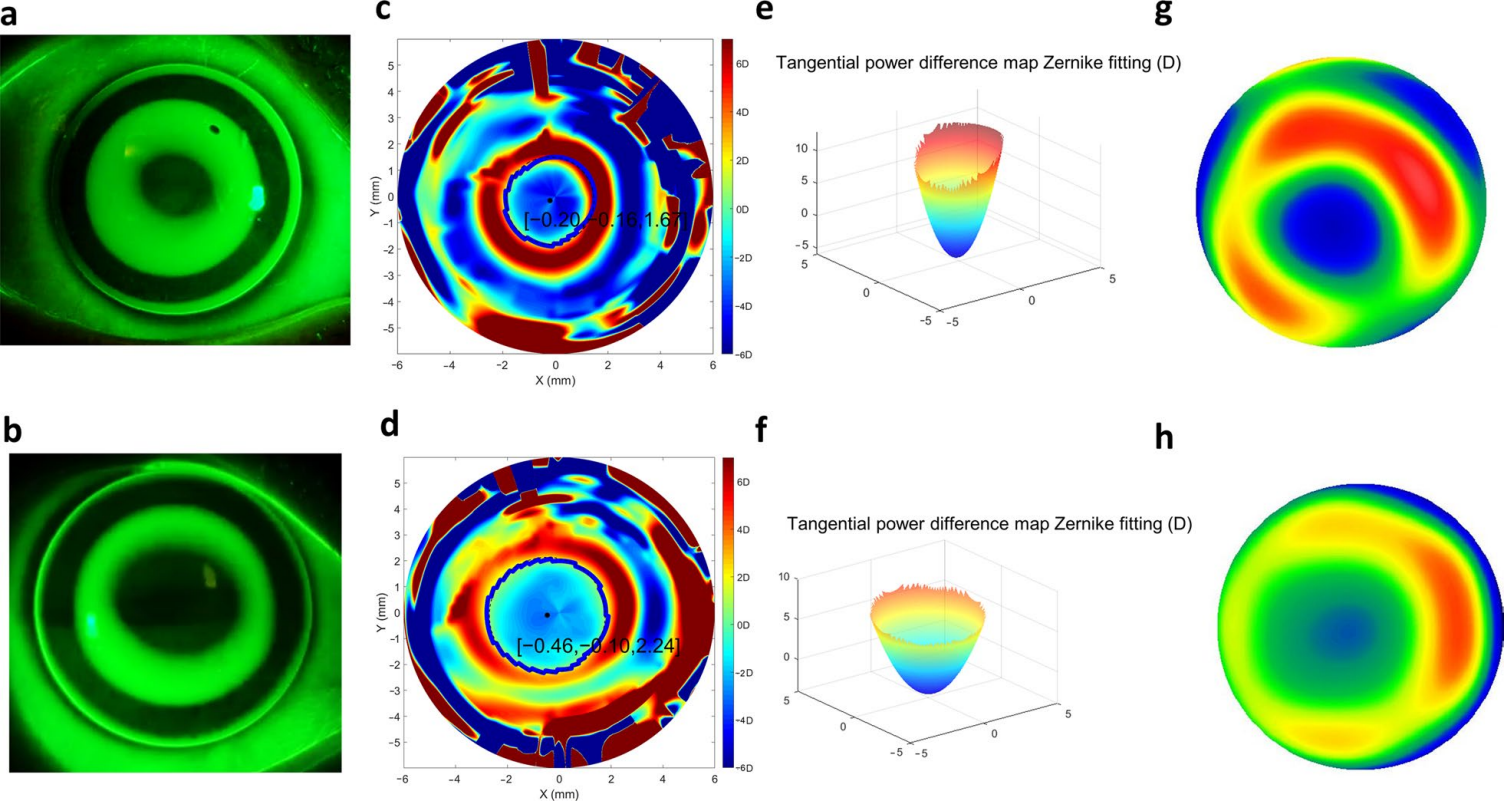
Two representative patients after one month of orthokeratology lens wearing with different back optic zone diameters (BOZDs). The upper row is patient A with a 5-mm BOZD, and the lower row is patient B with a 6-mm BOZD. Fluorescein pattern images of (**a**) patient A with 5-mm BOZD lenses and (**b**) patient B with 6-mm BOZD lenses. Clinical tangential power difference map of (**c**) patient A and (**d**) patient B showed that the radius of treatment size was 1.67 mm and 2.24 mm, respectively. Reproduction of the Zernike defocus coefficient in three-dimensional space of (**e**) patient A was 6.91 D and (**f**) patient B was 4.29 D. High-order aberrations of (**g**) patient A were 1.98 root mean square (RMS), and (**h**) patient B was 0.94 RMS. The baseline spherical equivalent (− 2.75 D) and age (9 years old) were similar, and the 12-month axial elongation was 0.05 and 0.34 mm, respectively

The TZ was automatically selected using a customized MATLAB program, in which the border was identified as the transition point from negative to positive values on the tangential difference map. The TZ area was calculated as $\pi \cdot {r}^{2}$, where r is the radius of the best-fitting ‘ring’ by the least-squares method for 256 points on the border of the TZ. The vertical and horizontal decentration of the TZ ([x, y]) can also be deduced from the ‘ring’, which are shown in Fig. 1c, d.

The calculation of the Zernike defocus coefficient (${C}_{2}^{0}$, D) also uses the raw data of corneal tangential power. Considering that the spatial defocus after ortho-K has different units in the three axes, we first normalized the horizontal and vertical directions and then used a customized MATLAB program to apply the following formula:

$${C}_{2}^{0}= \frac{1}{\pi }{\int }_{0}^{2\pi }{\int }_{0}^{1}{({\rm T}}_{post}\left(\rho ,\theta \right)-{\rm T}_{pre}\left(\rho ,\theta \right))\sqrt[2]{3}(2{\rho }^{2}-1)\rho d\rho d\theta$$, ($$\rho \epsilon \left[\mathrm{0,1}\right],\theta \epsilon [\mathrm{0,2}\pi ]$$)

In the formula, ${\rm T}$ is the matrix of corneal tangential power, $\rho$ is the radial defocus ring coordinate, and $\theta$ is the azimuthal angle. The border of the defocus ring is defined as the maximal positive defocus point on the difference map where ${\rm T}_{pre}\left(\rho ,\theta \right)$ and ${\rm T}_{post}\left(\rho ,\theta \right)$ are the matrices before and after ortho-K treatment, respectively, and the spherical defocus of Zernike polynomials [30] was used to fit the Zernike defocus coefficient (Fig. 1e, f). Zernike defocus coefficient means intensity of the tangential refractive power change on the cornea after ortho-K. In other words, after reshaping of the cornea, Zernike defocus coefficient characteristics tending to the ‘bowl’-like shape suggests good myopia control (Fig. 1e), while defocus characteristics tending to the ‘pan’-like shape means poor myopia control (Fig. 1f). Higher Zernike defocus coefficient indicates better myopia control effect.

### Statistical analysis

Only data from the right eyes of subjects who attended all scheduled visits were included in the analysis to avoid a strong correlation between right and left eye variables. All statistical analyses were performed using SPSS (v.26.0, IBM, Armonk, NY, USA). Descriptive statistics were calculated, and the means and standard deviations (SD) or numbers and percentages are reported as appropriate. The Shapiro‒Wilk test was performed to test the normality of the data. AL at baseline and at every visit was analyzed by repeated measures analysis of variance (ANOVA).

Since the reshaping of the eye by ortho-K reaches a stable level with no statistical significance within one month [29, 31, 32], we selected variables such as HOAs, TZ size and Zernike defocus coefficient at one month to be included in the analysis. The HOAs, TZ size and Zernike defocus coefficient values were compared using the unpaired *t*-test. Univariate and multivariate linear regression were used to further examine the relationship between ocular biometrics and AL elongation.

Based on a previous study, a cut-off of 0.20 mm/year [33] was used to define a slow group (≤ 0.2 mm/year) and a rapid group (> 0.2 mm/year). After the patients were divided into two groups according to axial growth in one year, univariate and multiple binary logistic regression were used to assess the predictive power of HOAs, TZ size and the Zernike defocus coefficient as well as to calculate the area under the receiver operating characteristic (ROC) curve. A two-tailed *P* value less than 0.05 was considered statistically significant.

## Results

A total of 320 subjects were assessed for eligibility, but six had a change in their lens prescription at the one month visit, and 13 others withdrew from the study due to time commitments; thus, only 301 subjects completed the study.

There were no statistically significant differences between different BOZD groups with regards to baseline characteristics (Table 1).

**Table 1:** Demographics and biometric data of ortho-K users at baseline

Parameter	6-mm group	5-mm group	*P* value
	(*n* = 147)	(*n* = 154)	
Age (years)	9.33 ± 1.77	9.67 ± 1.93	0.12
Male/female	72/75	82/72	0.46
AL (mm)	24.65 ± 0.76	24.68 ± 0.89	0.74
SER (D)	− 2.60 ± 1.24	− 2.86 ± 1.62	0.12
Ave-K (D)	43.34 ± 1.38	43.26 ± 1.85	0.67
PD (mm)	4.27 ± 0.58	4.36 ± 0.76	0.25
e value	0.54 ± 0.10	0.52 ± 0.11	0.08

*6 mm* = ortho-K lenses with a BOZD of 6 mm; *5 mm* = ortho-K lenses with a BOZD of 5 mm; *AL* = axial length; *SER* = spherical equivalent refractive error; *Ave-K* = average K reading; *PD* = pupil diameter

### Changes in VA

VA (recorded as logMAR) at 1, 6 and 12 months are shown in Additional file 1: Table S1. No significant difference in VA was observed between the groups (1 month, *P* = 0.23; 6 months, *P* = 0.19; 12 months, *P* = 0.15). There was also no correlation between TZ and VA (1 month, *P* = 0.260; 6 months, *P* = 0.560; 12 months, *P* = 0.363).

### Changes in AL

During the one-year follow-up period, repeated-measures ANOVA indicated that there was a significant difference between the two different BOZD groups in axial elongation (within subjects *P* < 0.001, between subjects *P* < 0.001).

**Fig. 2:**
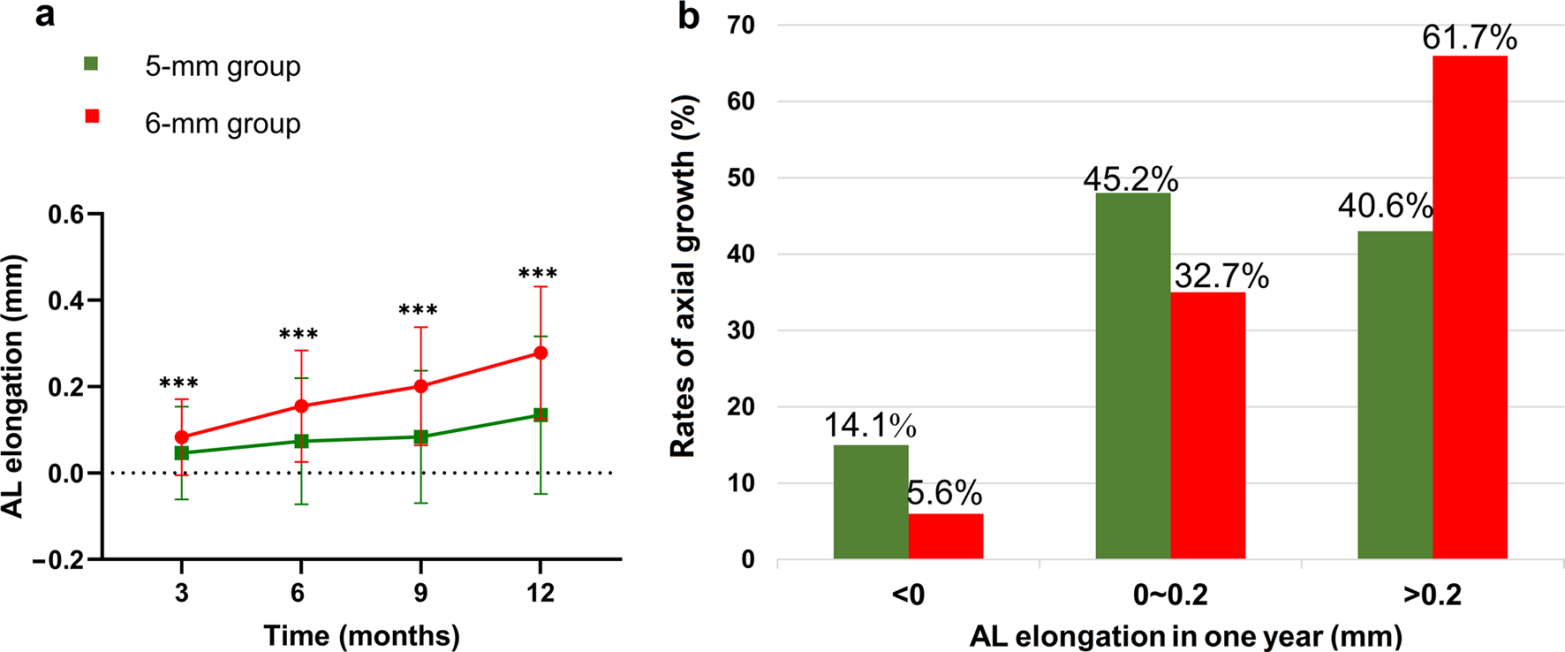
Axial elongation in one year based on the usage of different back optic zone diameter (BOZD). **a** The alterations in axial length (AL) within the 5-mm back optic zone diameter (BOZD) group were measured as 0.07 ± 0.15 mm during the 6-month examination and 0.13 ± 0.18 mm during the 12-month examination. These changes were comparatively lesser than those observed in the 6-mm BOZD group, which were recorded as 0.14 ± 0.13 mm during the 6-month visit and 0.27 ± 0.15 mm during the 12-month visit. (6-mm, 5-mm: the BOZD of the orthokeratology lenses measuring 6 mm and 5 mm, respectively; data are expressed as mean ± SD; repeated measures ANOVA, ^***^P < 0.001). **b** The occurrence rates of axial growth measurements falling below 0 mm, ranging between 0 and 0.2 mm, and exceeding 0.2 mm, in relation to various BOZDs of orthokeratology

As shown in Fig. 2a, AL changes in the 5-mm group were 0.07 ± 0.15 mm at the 6-month visit and 0.13 ± 0.18 mm at the 12-month visit; these changes were smaller than those of the 6-mm group, which were 0.14 ± 0.13 mm at the 6-month visit and 0.27 ± 0.15 mm at the 12-month visit. Additional file 1: Fig. S1 also showed the AL difference during follow-up visit. As shown in Fig. 2b, 14.1% of patients in the 5-mm group and 5.6% of subjects in the 6-mm group had less than 0 mm of axial elongation in one year. In the 5-mm group and 6-mm group, respectively, the prevalence of axial growth between 0 and 0.2 mm were 45.2% and 32.7%, while the prevalence of AL change greater than 0.2 mm were 40.6% and 61.7%.

### Changes in TZ, Zernike defocus coefficient and corneal HOAs

**Fig. 3:**
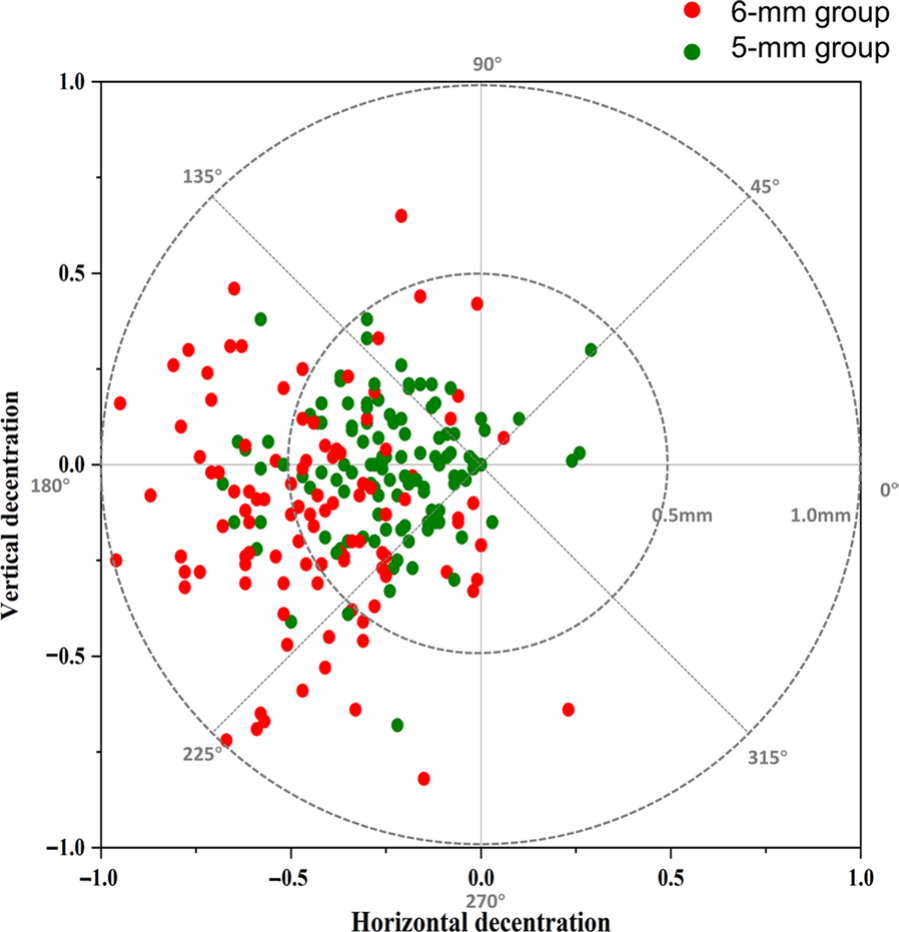
Decentration in eyes treated with different back optic zone diameter (BOZD) sizes

As shown in Fig. 1c, d, the mean TZ diameter was significantly smaller in the 5-mm group than in the 6-mm group at the one-month visit (3.41 ± 0.31 mm vs. 3.97 ± 0.38 mm, *P* < 0.001). Compared to the 5-mm group, the 6-mm group maintained increased horizontal decentration (− 0.23 mm vs. − 0.46 mm, respectively, *P* < 0.001) and vertical decentration (− 0.02 mm vs. − 0.11 mm, respectively, *P* < 0.001), as shown in Fig. 3. A significantly higher Zernike defocus coefficient (unpaired *t*-test, *P* < 0.001) was found in the 5-mm group (4.97 ± 1.54 D) than in the 6-mm group (4.40 ± 1.62 D), which implies higher defocus in 5-mm BOZD eyes.

Additionally, at the one-month time point, the 5-mm group showed a significant increase in the difference in total HOAs, and total SA compared with the 6-mm group but not in total coma. The results also showed that coma II Z(5, ± 1), coma III Z(7, ± 1), spherical aberration Z(4, ± 0), and spherical aberration II Z(6, ± 0) were the main sources of differences between the 5-mm and 6-mm groups (all *P* < 0.05, Additional file 1: Table S2).

### Univariate and multivariate linear regression

To analyze factors affecting AL elongation, parameters including age, sex, SER, PD, e value, Ave-K, decentration, TZ size, Zernike defocus coefficient and HOAs were included in the regression analysis. In Additional file 1: Table S3, the univariate analysis showed significant correlations with age (beta =  − 0.031, *P* < 0.001), SER (beta = 0.041, *P* < 0.001), e value (beta = 0.284, *P* < 0.008), horizontal decentration (beta =  − 0.212, *P* < 0.001), TZ size (beta = 0.281, *P* < 0.001) and HOAs (beta =  − 0.045, *P* < 0.024). After adjusting for potential confounders (Horizontal decentration and HOAs) in the multivariable regression models, older age (beta =  − 0.024, *P* < 0.001), more myopia (beta = 0.027, P = 0.003), e value (beta = 0.223, *P* < 0.038) and smaller TZ size (beta = 0.186, *P* = 0.002) were significantly associated with less AL elongation in multivariate linear regression analyses.

### Univariate and multiple binary logistic regression and ROC curve

Univariate logistic regression analysis (Additional file 1: Table S4) showed that older age (OR = 0.713, *P* = 0.002), male sex (OR = 0.484, *P* = 0.046) and small TZ size (OR = 62.578, *P* < 0.001) were correlated with slow AL elongation. Multifactorial logistic regression analysis was conducted based on univariate analyses. Older age (OR = 0.706, P < 0.001) and smaller TZ diameter (OR = 5.121, *P* < 0.001) were still protective factors against AL elongation except sex (*P* = 0.138). As shown in Additional file 1: Table S5, the binary logistic regression analysis showed that the equation was as follows: ${Ln}^{\frac{\mathrm{Y}}{1-Y}}$ = − 2.84 − 0.352 × X2 + 1.690 × X3 (X2 = age; X3 = TZ diameter), while “Y” stands for the likelihood of rapid axial elongation.

**Fig. 4:**
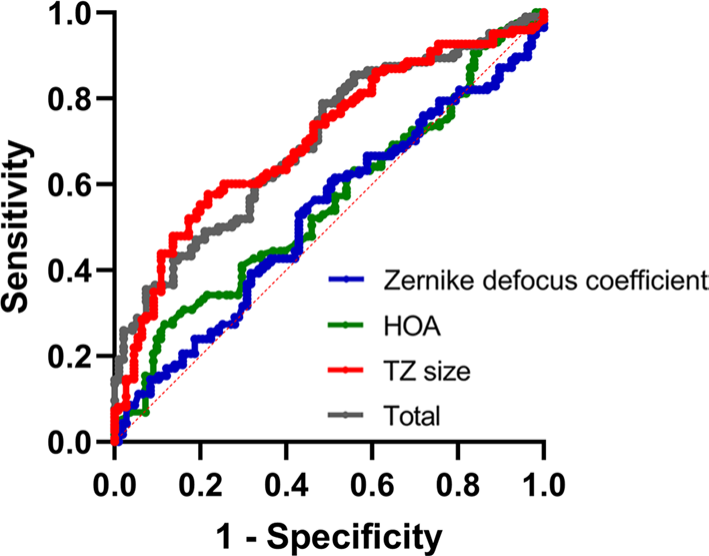
Receiver operating characteristic (ROC) curves for predicting rapid (> 0.2 mm/year) or slow (≤ 0.2 mm/year) axial elongation. HOA, high-order aberration; TZ, treatment zone; Total, equation combining the Zernike defocus coefficient, HOA and TZ size

To predict AL elongation, ROC analysis revealed that TZ size yielded a larger area under the ROC curve (AUC), at 0.684 (95% CI, 0.610 to 0.758, *P* < 0.001), than the Zernike defocus coefficient of 0.560 (95% CI, 0.480 to 0.641, *P* = 0.141) or HOAs of 0.584 (0.505 to 0.664, *P* = 0.037) (Fig. 4). The AUC of the combined ROC analysis of TZ diameter, Zernike defocus coefficient and HOAs was 0.699 (95% CI, 0.627 to 0.771, *P* < 0.001), which was slightly greater than the value achieved using TZ size alone (Fig. 4 grey). The cut-off value of TZ diameter was defined as 3.82 mm.

## Discussion

Our study prospectively shows that the 5-mm BOZD ortho-K could better control myopia than the 6-mm BOZD ortho-K. Comparing the changes between the two different BOZD groups, our study found that the 5-mm group had a smaller TZ diameter, less decentration, more defocus and higher HOAs. We also attempted to identify factors that affect the progression of myopia and found that age and TZ diameter affected axial elongation, both in linear regression and in logistic regression. TZ diameter was the best predictor of one-year axial elongation when divided into rapid (AL changes > 0.2 mm) and slow (AL changes ≤ 0.2 mm) groups. If the TZ diameter is less than 3.82 mm, there is a 70% probability that the axial length of the patient will change less than 0.2 mm in one year.

5-mm BOZD ortho-K had better control effects on axial elongation than the 6-mm BOZD ortho-K. Our research shows that the difference in axial elongation between the 6-mm group and the 5-mm group after one year of follow-up is 0.14 mm, which means that the control effect of the 5-mm group is 51.8% better than that of the 6-mm group. In addition, we also calculated the proportion of AL elongation, and the results showed that 38.3% of patients in the 6-mm group and 59.3% of patients in the 5-mm group had axial growth of 0.2 mm or less in one year, indicating that a higher proportion of patients with 5-mm BOZD ortho-K could achieve superior control effects.

Similar results were reported by Li et al. [25]; compared to the 6.2-mm BOZD group (Euclid), the 5-mm BOZD group (DRL) can reduce axial elongation by 0.15 mm/year (53.6%). Guo et al. [24] reported in a randomized controlled trial (RCT) that found that 5-mm BOZD ortho-K (KATT BE Free, Precision Technology Services) compared with 6-mm BOZD can reduce axial elongation by 0.13 mm (76.5%). On the other hand, Pauné et al.[34] observed that smaller BOZD ortho-K (double reservoir lens design, DRL) can reduce myopia progression by approximately 0.06 mm/year (40%). Despite the different amounts of AL elongation partly caused by different lens designs and inclusion criteria, various studies have shown that reducing the BOZD can improve the effectiveness of myopia control.

Higher HOAs induced by smaller BOZDs ortho-K may be a potential factor for slow AL growth [8, 35]. In this study, we saw that a smaller BOZD contributed to higher total HOAs and total SA. Carracedo et al. [23] proved that after 5-mm BOZD, eyes showed a greater corneal spherical aberration after two weeks of ortho-K wearing, while Li et al. [25] proposed that smaller BOZD contributes to the increase in horizontal coma but not SA after one year. We did not observe any differences in coma, possibly due to lens decentration being the main cause of coma and the decentration of 5-mm BOZD was relatively small. Although the difference in Zernike components was inconsistent in different BOZD lenses which are greatly influenced by different methods and diameter measurement ranges [22], the total HOAs in smaller BOZDs is higher than that in larger BOZDs.

Our current results support the view that a smaller TZ may lead to better myopia control, which has been confirmed by several studies [24, 34, 36, 37]. Furthermore, our study creatively proposed that a TZ diameter at the one-month visit of less than 3.82 mm was the cut-off value to predict changes in AL of less than 0.2 mm in one year, which may improve our efficiency in judging the effectiveness of myopia control. In fact, during the early visit, we can judge whether it is necessary to adjust the personalized myopia control plan, for example, combined with low concentration of atropine for better control effect. It is worth noting that although the definitions of TZ boundaries are similar in the different studies, which range from negative to positive values on the topography maps, except for the lens design, different topographers and different methods to measure TZ parameters [24, 38–40] are some of the reasons for the different TZ sizes in the results. When faced with different lens designs and using different methods of measuring TZ, the effective size of the TZ for controlling axial elongation needs to be further explored. Customized algorithms can be built into corneal topographic maps to facilitate clinical judgment but this needs further study.

Ortho-K lens decentration affects myopic control effect by changing peripheral defocus on the retina. Ortho-K lens decentration is common in clinical practice and may be induced by lens fitting, lens diameter, and corneal toricity [41–43], but the influence of ortho-K lens decentration on AL elongation remains controversial. Sun et al. [44] reported that there was no significant difference in subjects with different TZ decentration, while Li et al. [45] proposed that the greater the decentration of TZ, the slower the axial growth, in which TZ decentration caused local defocusing changes to inhibit myopic progression. Similar results were also proposed by Zhang et al. [46] and Chu et al. [47]. The research by Lin et al. [48] has proven that TZ decentration can cause larger summed relative corneal refractive power, potentially affecting myopic defocus on the retina. In our study, the 5-mm group had less TZ decentration but less axial elongation. When we reduce the size of BOZD, a smaller TZ diameter may bring a wider and steeper peripheral ring and more defocus than decentration only e.g., after wearing 5-mm BOZD ortho-K lenses to reshape the cornea, the Zernike defocus coefficient was higher than that after wearing 6-mm BOZD lenses. While there was no significant difference in pupil size between the two groups (4.27 mm vs. 4.35 mm, *P* = 0.295), more defocus on the cornea may create a more effective peripheral myopic defocus on the retina to achieve a better control effect.

The less TZ decentration in the 5-mm group was closely related to the design of the lenses. With the similar lens diameter (5-mm group: 10.87 mm, 6-mm group: 10.80 mm, *P* > 0.05, unpaired *t*-test), 5-mm BOZD lenses have a lower return zone depth (RZD) (approximately 92 μm) to achieve a smaller BOZD, (5-mm group: 438.5 μm, 6-mm group: 530.8 μm, *P* < 0.05, unpaired *t*-test) and landing zone angle (LZA) corresponds to flatter (5-mm group: 31.2°, 6-mm group: 31.9°, *P* < 0.05, unpaired *t*-test). Due to the lower RZD design of the 5-mm BOZD lenses, the actual contact area between LZA and the cornea is larger, which is a possible reason for the better positioning of 5-mm lenses.

The relationship between TZ size, higher Zernike defocus coefficient, and HOA after corneal reshaping and pupil size is also an important factor affecting effective peripheral retinal myopia defocus on the retina [49]. One of the limitations of our study is that we did not discuss the impact of these three factors on myopia control effectiveness within the pupil. However, when we divided participants by pupil size (large pupil ≥ 4.28 mm, small pupil < 4.28 mm, divided by average pupil size), we found that there was no significant difference in axial elongation between the large pupil group and the small pupil group with the same BOZD (Additional file 1: Table S5). A possible explanation is that there is a large bias in pupil measurement, and the instantaneous scotopic pupil size measured in the examination room is not sufficient to reflect the scotopic pupil size of children during daily activities in different environments. Therefore, in our study, pupil size was not a factor affecting AL elongation.

In addition, although a smaller TZ size is better for myopia control, we should also pay attention to balancing visual quality and myopia progression. In our study, six patients were out of the study due to prescription lens replacement, three of whom complained of poor vision. The threshold value of TZ size that can ensure both comfortable vision and a good control effect needs further research. The study also did not consider previous myopia history of participants, family history of myopia, time outdoors, nutrition and the economic situation which may result in bias.

## Conclusion

Compared to the 6-mm group, the 5-mm group showed a retardation of axial elongation by 0.14 mm (51.8%). A smaller BOZD showed a smaller TZ size, higher Zernike defocus coefficient and higher HOA on the cornea. TZ size was a better predictor of AL elongation, and a TZ with a diameter of less than 3.82 mm may lead to AL elongation of less than 0.2 mm in one year.

*What is already known on this topic*—Decreasing the back optic zone diameter (BOZD) of orthokeratology lenses may reduce the size of the treatment zone, providing a protective effect against axial elongation.

*What this study adds*—We report the efficacy of a small back optic zone diameter (BOZD) design for corneal refractive therapy (CRT). Decreasing the BOZD by increasing higher-order aberrations (HOAs), raising the Zernike defocus coefficient (meaning steeper peripheral defocus) and reducing the treatment zone (TZ) size increased the effectiveness of myopia control. Furthermore, we found that TZ size was the best factor for predicting myopia control, and a TZ diameter of less than 3.82 mm was associated with an axial elongation rate of less than 0.2 mm/year.

*How this study might affect research*—Our research further validates the reason that smaller BOZDs produce better myopia control effects and proposes a TZ size threshold for an improved effect, which is helpful for efficient myopia control.

### Supplementary Information

**Additional file 1: Table S1. **Visual acuity (logMAR) after orthokeratology at 1 month, 6 months and 12 months visits. **Table S2.** Individual Zernike coefficients and root mean square (RMS) of high-order aberration in different BOZD eyes. **Table S3.** Liner regression analyses of 12-month axial length elongation and ocular parameters. **Table S4.** Logistic regression analysis of axial length (AL) elongation and ocular parameters. **Table S5.** Axial elongation in one year based on the size of the pupil and the usage of different BOZD. **Figure S1.** Axial length changes in one year based on the usage of different back optic zone diameter (BOZD). Axial length changes over 12 months of follow-up in the two groups of ortho-K users. 6-mm, 5-mm, the BOZD of the ortho-K lenses measured 6 and 5 mm, respectively; AL, axial length. Data are expressed as the mean ± SD; Repeated-measures ANOVA was used, *P<0.05; **P<0.01).

## Data Availability

The data that support the findings of this study are available from the corresponding author upon reasonable request.
