# Supplementary material for: Efficacy of small back optic zone design on myopia control for corneal refractive therapy (CRT): a one-year prospective cohort study
Source: Eye Vis (Lond). 2023 Nov 20;10:47. doi: 10.1186/s40662-023-00364-z (PMC10658859; doi:10.1186/s40662-023-00364-z)
Supplement: Supplementary file 1 — Additional file 1: Table S1. Visual acuity (logMAR) after orthokeratology at 1 month, 6 months and 12 months visits. Table S2. Individual Zernike coefficients and root mean square (RMS) of high-order aberration in different BOZD eyes. Table S3. Liner regression analyses of 12-month axial length elongation and ocular parameters. Table S4. Logistic regression analysis of axial length (AL) elongation and ocular parameters. Table S5. Axial elongation in one year based on the size of the pupil and the usage of different BOZD. Figure S1. Axial length changes in one year based on the usage of different back optic zone diameter (BOZD). Axial length changes over 12 months of follow-up in the two groups of ortho-K users. 6-mm, 5-mm, the BOZD of the ortho-K lenses measured 6 and 5 mm, respectively; AL, axial length. Data are expressed as the mean ± SD; Repeated-measures ANOVA was used, *P<0.05; **P<0.01). [file 40662_2023_364_MOESM1_ESM.docx]

**Additional File**

**Table S1.** Visual acuity (logMAR) after orthokeratology at 1 month, 6 months and 12 months visits

| Time | 6-mm group | 5-mm group | *P* value |
| --- | --- | --- | --- |
| 1 month | −0.01±0.08 | 0.00±0.08 | 0.23 |
| 6 months | −0.02±0.06 | −0.01±0.07 | 0.19 |
| 12 months | −0.02±0.06 | −0.01±0.08 | 0.15 |

6‐mm = orthokeratology lenses of BOZD 6 mm; 5‐mm = orthokeratology lenses of BOZD 5 mm; Unpaired‐t tests for difference between groups, mean ± standard deviation

**Table S2.** Individual Zernike coefficients and root mean square (RMS) of high-order aberration in different BOZD eyes

| Parameters | 6-mm group |  |  | 5-mm group |  |  | *P* value |
| --- | --- | --- | --- | --- | --- | --- | --- |
|  | Baseline | 1 month | **Difference** | Baseline | 1 month | **Difference** |  |
| Total HOAs RMS (μm) | 0.4317±0.1999 | 1.5624±0.7320 | 1.1307±0.7397 | 0.4541±0.1684 | 1.6079±0.5308 | 1.2948±0.4346 | **0.034** |
| Total SA RMS (μm) | 0.2003±0.1862 | 0.9950±0.4372 | 0.7947±0.4339 | 0.2048±0.1142 | 1.1263±0.3861 | 0.9215±0.3861 | **0.010** |
| Total Coma RMS (μm) | 0.2982±0.1371 | 0.9204±0.6781 | 0.5696±0.5737 | 0.3148±0.1110 | 0.9361±0.4497 | 0.6026±0.3756 | 0.567 |
| Trifoil *Z(3,±3)* (μm) | 0.1865±0.0953 | 0.2944±0.2246 | 0.1079±0.2469 | 0.2153±0.1310 | 0.3112±0.2093 | 0.1031±0.2428 | 0.879 |
| Coma *Z(3,±1)* (μm) | 0.2469±0.1325 | 0.9338±0.6514 | 0.6869±0.6646 | 0.2348±0.1430 | 0.8527±0.4502 | 0.7140±0.4251 | 0.702 |
| Quadrifoil *Z(4,±4)* (μm) | 0.0864±0.0578 | 0.1435±0.0983 | 0.0570±0.1024 | 0.0909±0.0575 | 0.1444±0.1162 | 0.0637±0.1214 | 0.644 |
| Astigmatism II *Z(4,±2)* (μm) | 0.0647±0.0503 | 0.2555±0.2367 | 0.1171±0.8580 | 0.0810±0.0511 | 0.2908±0.1600 | 0.2360±0.1505 | 0.127 |
| Spherical aberration *Z(4,±0)* (μm) | 0.2068±0.1888 | 1.0117±0.3880 | 0.8050±0.4069 | 0.2053±0.1120 | 1.0928±0.3874 | 0.9818±0.3395 | **<0.001** |
| Pentafoil *Z(5,±5)* (μm) | 0.0595±0.0290 | 0.1038±0.0685 | 0.0443±0.0769 | 0.0617±0.0344 | 0.1067±0.0792 | 0.0540±0.0838 | 0.342 |
| Trifoil II *Z(5,±3)* (μm) | 0.0487±0.0326 | 0.1231±0.0997 | 0.0745±0.1060 | 0.0573±0.0355 | 0.1344±0.1134 | 0.0898±0.1203 | 0.288 |
| Coma II *Z(5,±1)* (μm) | 0.0395±0.0389 | 0.1821±0.2051 | 0.1129±0.4152 | 0.0485±0.0427 | 0.3035±0.2020 | 0.2794±0.1995 | **<0.001** |
| Esafoil *Z(6,±6)* (μm) | 0.0306±0.0170 | 0.0716±0.0601 | 0.0410±0.0610 | 0.0342±0.0221 | 0.0659±0.0591 | 0.0367±0.0630 | 0.591 |
| Quadrifoil II *Z(6,±4)* (μm) | 0.0320±0.0302 | 0.0636±0.0708 | 0.0242±0.1128 | 0.0294±0.0170 | 0.0517±0.0558 | 0.0251±0.0634 | 0.941 |
| Astigmatism III *Z(6,±2)* (μm) | 0.0203±0.0157 | 0.1036±0.0874 | 0.0832±0.0883 | 0.0240±0.0199 | 0.1049±0.0851 | 0.0902±0.0902 | 0.542 |
| Spherical aberration II *Z(6,±0)* (μm) | 0.0099±0.0226 | -0.0528±0.1485 | 0.0627±0.1501 | 0.0094±0.0309 | 0.1671±0.1825 | 0.1636±0.1830 | **<0.001** |
| Eptafoil *Z(7,±7)* (μm) | 0.0221±0.0364 | 0.0460±0.0463 | 0.0266±0.0441 | 0.0210±0.0137 | 0.0505±0.0417 | 0.0327±0.0441 | 0.243 |
| Pentafoil II *Z(7,±5)* (μm) | 0.0309±0.0515 | 0.0416±0.0297 | 0.0107±0.0612 | 0.0248±0.0172 | 0.0403±0.0372 | 0.0206±0.0425 | 0.140 |
| Trifoil III *Z(7,±3)* (μm) | 0.0341±0.0164 | 0.0591±0.0696 | 0.0394±0.0697 | 0.0207±0.0143 | 0.0533±0.0411 | 0.0376±0.0423 | 0.438 |
| Coma III *Z(7,±1)* (μm) | 0.0182±0.0274 | 0.1323±0.0965 | 0.1143±0.0951 | 0.0153±0.0136 | 0.1333±0.0802 | 0.1487±0.0780 | **0.002** |

HOAs = high-order aberrations, 3rd order to 7th order; BOZD = back optic zone diameters; SA = spherical aberration; total SA = square root of the sum of the squared coefficients of *Z(4,0)*and *Z(6,±0)*; total Coma = square root of the sum of the squared coefficients of *Z(3,±1)*and *Z(5,±1)*; Unpaired t-test, mean ± standard deviation; Bold typeface values indicate statistical significance

**Table S3.** Liner regression analyses of 12-month axial length elongation and ocular parameters

| Parameters | Univariate regression | | Multivariate regression | |
| --- | --- | --- | --- | --- |
|  | Beta (95% CI) | *P* value | Beta (95% CI) | *P* value |
| Age (years) | −0.031 (−0.042 to −0.020) | **<0.001** | −0.024 (−0.035 to −0.012) | **<0.001** |
| Gender (boys) | 0.039 (−0.006 to 0.084) | 0.088 | − | − |
| SER (D) | 0.041 (0.025 to 0.057) | **<0.001** | 0.027 (0.010 to 0.044) | **0.003** |
| Ave K (D) | 0.007 (−0.09 to 0.023) | 0.386 | − | − |
| PD (mm) | −0.006 (−0.039 to 0.027) | 0.710 | − | − |
| e value | 0.284 (0.074 to 0.494) | **0.008** | 0.223 (0.012 to 0.433) | **0.038** |
| Horizontal decentration (mm) | −0.212 (−0.306 to −0.117) | **<0.001** | −0.031 (−0.145 to 0.083) | 0.591 |
| Vertical decentration (mm) | 0.073 (−0.029 to 0.174) | 0.159 | − | − |
| TZ size (mm) | 0.281 (0.182 to 0.380) | **<0.001** | 0.186 (0.069 to 0.303) | **0.002** |
| Zernike defocus coefficient (D) | −0.009 (−0.024 to 0.006) | 0.259 | − | − |
| HOAs (RMS) | −0.045 (−0085 to −0.06) | **0.024** | −0.057 (−0.065 to 0.180) | 0.357 |

CI = confidence interval; D = diopter; Ave-K = corneal average K reading; PD = pupil diameter; SER = spherical equivalent refraction; AL = axial length; TZ = treatment zone; HOAs = high-order aberrations; RMS = root mean square; Bold typeface values indicate statistical significance

**Table S4** Logistic regression analysis of axial length (AL) elongation and ocular parameters

| Parameters | Univariate regression | | Multivariate regression | |
| --- | --- | --- | --- | --- |
|  | OR (95% CI) | *P* value | OR (95% CI) | *P* value |
| Age (years) | 0.713 (0.578 to 0.880) | **0.002** | 0.706 (0.599 to 0.833) | **<0.001** |
| Gender (boys) | 0.484 (0.237 to 0.986) | **0.046** | 1.546 (0.869 to 2.750) | 0.138 |
| SER (D) | 1.356 (0.993 to 1.852) | 0.055 | − | − |
| Ave K (D) | 0.954 (0.747 to 1.220) | 0.710 | − | − |
| PD (mm) | 0.933 (0.544 to 1.600) | 0.802 | − | − |
| e value | 8.825 (0.896 to 86.891) | 0.062 |  |  |
| Horizontal decentration (mm) | 3.433 (0.512 to 23.013) | 0.204 | − | − |
| Vertical decentration (mm) | 2.183 (0.415 to 11.487) | 0.357 | − | − |
| TZ size (mm) | 5.326 (2.710 to 10.467) | **<0.001** | 5.121 (2.522 to 10.402) | **<0.001** |
| Zernike defocus coefficient (D) | 1.206 (0.907 to 1.604) | 0.197 | − | − |
| HOAs (RMS) | 0.543 (0.261 to 1.132) | 0.103 | − | − |

CI = confidence interval; OR = odds ratio; D = diopter; Ave-K = corneal average K reading; PD = pupil diameter; SER = spherical equivalent refraction; AL = axial length; TZ = treatment zone; HOAs = high-order aberrations; RMS = root mean square; Bold typeface values indicate statistical significance

**Table S5**. Axial elongation in one year based on the size of the pupil and the usage of different BOZD.

| Parameter | AL elongation with large pupil  (PD ≥ 4.28 mm) | AL elongation with small pupil (PD < 4.28 mm) | *P* value |
| --- | --- | --- | --- |
| 5-mm BOZD group | 0.17±0.17 mm | 0.14±0.20 mm | 0.696 |
| 6-mm BOZD group | 0.26±0.19 mm | 0.24±0.16 mm | 0.877 |
| *P* value | <0.05 | <0.05 | － |

AL = axial length; PD = pupil diameter; BOZD = back optic zone diameter; Unpaired t test.

**Figure S1**. Axial length changes in one year based on the usage of different back optic zone diameter (BOZD). Axial length changes over 12 months of follow-up in the two groups of ortho-K users. 6-mm, 5-mm, the BOZD of the ortho-K lenses measured 6 and 5 mm, respectively; AL, axial length. Data are expressed as the mean ± SD; Repeated-measures ANOVA was used, **P*<0.05; ***P*<0.01)

**
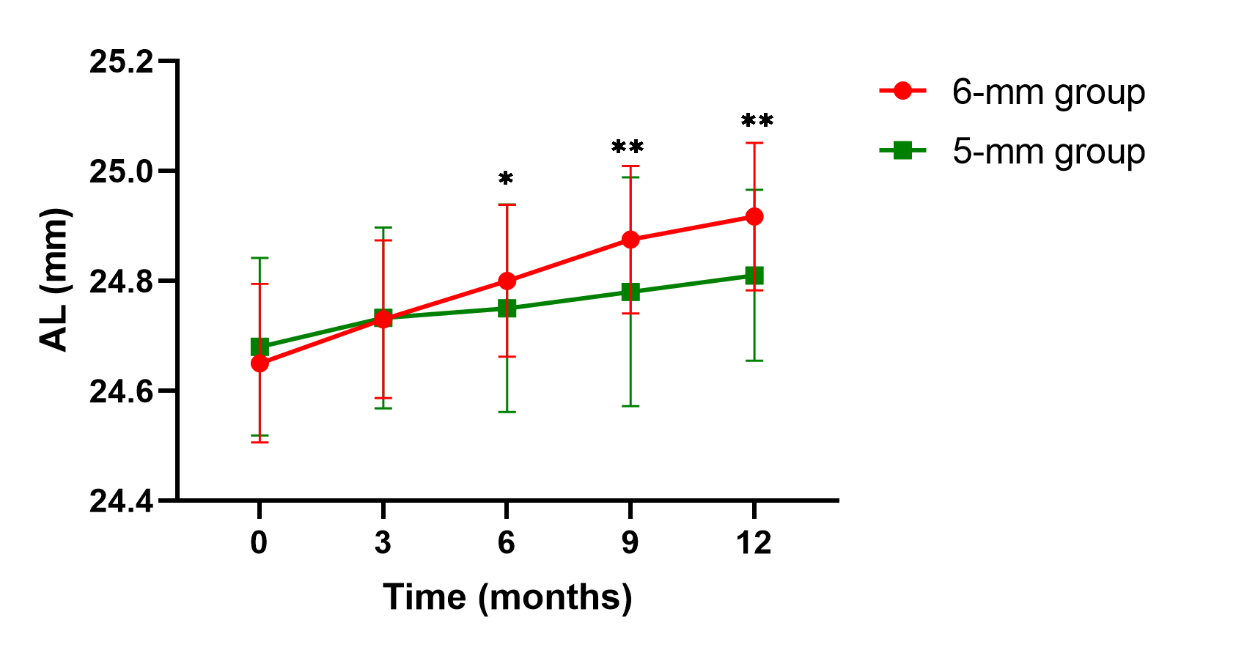
**
